# ShRNA knock‐down of CXCR7 inhibits tumour invasion and metastasis in hepatocellular carcinoma after transcatheter arterial chemoembolization

**DOI:** 10.1111/jcmm.13119

**Published:** 2017-04-21

**Authors:** Zhong‐Wei Zhao, Xiao‐Xi Fan, Jing‐Jing Song, Min Xu, Min‐Jiang Chen, Jian‐Fei Tu, Fa‐Zong Wu, Deng‐Ke Zhang, Lu Liu, Li Chen, Xi‐Hui Ying, Jian‐Song Ji

**Affiliations:** ^1^ Department of Radiology the Fifth Affiliated Hospital of Wenzhou Medical University Lishui Hospital of Zhejiang University Lishui Central Hospital Lishui China

**Keywords:** hepatocellular carcinoma, transcatheter arterial chemoembolization, lentiviral vector, shRNA, CXCR7, invasion, metastasis

## Abstract

To investigate the effects of lentiviral vector‐mediated shRNA suppressing CXCR7 on tumour invasion and metastasis in hepatocellular carcinoma (HCC) after transcatheter arterial chemoembolization (TACE). HCCLM3 cell lines were cultured and assigned into the CXCR7‐shRNA, negative control (NC) and blank groups. The qRT‐PCR and Western blotting were applied to detect the mRNA and protein expressions of CXCR7, CXCR4 and MMP‐2 in HCCLM3 cells. Cell proliferation and invasion were evaluated by MTT and Transwell assays. A Buffalo rat model of HCC was established. Fifty model rats were divided into the CXCR7‐shRNA + TACE, CXCR7‐shRNA, TACE, NC and control groups. Immunohistochemistry was performed to detect the expressions of CXCR7, MMP‐2, vascular endothelial growth factor (VEGF) and intratumoral CD31‐positive vessel count in tumour tissues of mice. Compared with the blank and NC groups, the mRNA and protein expressions of CXCR7 and MMP‐2 were decreased in the CXCR7‐shRNA group. The cell proliferation and invasion rates of the CXCR7‐shRNA group were lower than the blank and NC groups. At the 4th week after TACE, tumour weight of the *CXCR7*‐shRNA + TACE group increased continuously. The *CXCR7*‐shRNA + TACE group showed longer survival time and smaller tumour sizes than other groups. Compared with other groups, the CXCR7‐shRNA + TACE and CXCR7‐shRNA groups had less number of lung metastatic nodules and lower expressions of CXCR7, MMP‐2, VEGF and CD31‐positive vessel count. CXCR7‐shRNA inhibits tumour invasion and metastasis to improve the efficacy of TACE in HCC by reducing the expressions of CXCR7, MMP‐2 and VEGF.

## Introduction

Primary liver cancer is the sixth most common cancer worldwide, and the third and sixth most common causes of cancer‐related mortality 1, 2. Hepatocellular carcinoma (HCC) accounts for 85–90% of all liver cancers 3. A high incidence rate of HCC is reported in China, where 55% of the world's HCC cases occur 2. The risk factors for HCC include alcohol consumption, obesity, diabetes and metabolic syndromes, in addition to chronic hepatitis B virus (HBV) and/or hepatitis C virus (HCV) infections 4, 5, 6. Due to an absence of symptoms, HCC is frequently diagnosed in its late stages, when large tumours, unresectable lesions and poor liver function are present 7. One of the major challenges in improving the survival rate of patients with HCC is metastasis which is organ specific, with 30% of them being in the lungs 8.

Chemokines and their receptors have been reported to play essential roles in the chemotaxis of tumour cells to target organs 9. Therefore, the identification of metastasis‐related chemokine receptors can provide tumour therapy with potential targets. The chemotactic cytokines, also referred to as chemokines, comprise a superfamily of small, secreted cytokines, which were initially characterized by their ability to promote leucocyte migration 10. The chemokine receptors expressed in cancer cells have been indicated to play a role in cancer cell metastasis and migration 11. CXC chemokine receptor 7 (CXCR7), now classified as a chemokine receptor able to bind the chemokines CXCL12/SDF‐1 and CXCL11, was found to function primarily by sequestering the chemokine CXCL12, and is highly expressed in the HCC 12, 13, 14.

Potential curative treatments, including radiofrequency (RF) tissue ablation, surgical resection and liver transplantation, have been considered the most effective HCC therapy. However, they have been performed for fewer than 20% of patients with HCC, mainly because of complicating cirrhosis and/or an advanced stage of cancer at diagnosis 15. Transcatheter arterial chemoembolization (TACE), following its introduction in 1974, has become an established treatment for intermediate‐stage HCC (traditionally defined as Barcelona Clinic Liver Cancer B disease) 16, 17. The liver parenchyma primarily receives its blood supply from the portal vein, whereas the tumour predominantly receives blood from the hepatic artery. TACE enables the selective cannulation of vessels that supply the tumour and occlusion by embolic particles. This can lead to tumour hypoxia and necrosis 18. Although TACE is an effective treatment for HCC, it is not considered a curative procedure, and among the factors potentially weakening its effectiveness is a hypothetical neoangiogenic reaction due to ischaemia 19. As a palliative treatment modality, TACE also has several limitations. It can result in an incomplete necrosis of hypovascular or large tumours and may, therefore, demand repeated treatment periods. This can taper off the tumour‐feeding artery and deteriorate liver function 20, 21. Improvements in the effectiveness of TACE appear to be essential to TACE‐treated HCC. In the present study, we aimed to determine the effects of lentiviral vector‐mediated shRNA knock‐down of CXCR7 on tumour invasion and metastasis in patients with HCC after TACE.

## Materials and methods

### Construction of CXCR7‐shRNA lentiviral vector

A short hairpin RNA (shRNA) construct was designed targeting a 19‐nt sequence (5′‐GACACGGTGATGTGTCCCA‐3′) within the human CXCR7 gene (Gene ID: 57007). Table 1 presents the transcription templates of shRNA, in which TTCAAGAGA was selected for the stem‐loop‐shaped structure, and the T6 structure for the termination sequence for shRNA transcription. CCGG was added to the 5′ end of the positive‐sense primer template to complement the cut of the AgeI enzyme (sense sequence). AATT was added to the 5′ end of the negative‐sense primer template to complement the cut of the EcoRI enzyme (antisense sequence). pMagic 7.1 interference vector was applied for the construction of the *CXCR7*‐shRNA expression vector and was cut by both AgeI and EcoRI enzymes for linearization, followed by standing overnight at 16°C with T4 DNA ligase, the annealed double‐stranded oligo, and the transformation of Escherichia coli DH5α competent cells. For these, the recombinants of positive colonies were selected for polymerase chain reaction (PCR) and sequencing. The pMagic 7.1‐*CXCR7* recombinant vector, pLP1 vector, pLP2 vector and pLP/VSVG vector were subsequently mixed (Institute of Biochemistry and Cell Biology, SIBS, CAS) and transfected using a liposome as a mediator. They were then packed into lentiviral vector particles. The shRNA lentivirus expression vector of the negative control sequence was provided by Shanghai SBO Medical Biotechnology Co., Ltd., Shanghai, China.

**Table 1:** shRNA sequence information

Sense sequence	5′‐CCGGGACACGGTGATGTGTCCCATTCAAGAGATGGGACACATCACCGTGTCTTTTTTG‐3′
Anti‐sense sequence	5′‐AATTCAAAAAAGACACGGTGATGTGTCCCATCTCTTGAATGGGACACATCACCGTGT‐3′

### Cell culture and transfection

Human HCC cell line HCCLM3 was purchased from the Liver Cancer Institute, Zhongshan Hospital, Fudan University (Shanghai, China). The cells were cultured in a RPMI 1640 medium, supplemented with 10% foetal bovine serum, at 37°C with 5% CO_2_. The HCCLM3 cells in the logarithmic growth phase were assigned into three groups: the CXCR7‐shRNA group (transfected with LV‐CXCR7‐shRNA vector), the negative control (NC) group (transfected with LV‑shRNA negative control lentiviral vector) and the blank group. The transfection efficiency of the cells was subsequently detected under a fluorescence microscope, and the cells with the transfection efficiency over 80% were selected as target cells.

### Quantitative real‐time polymerase chain reaction (qRT‐PCR)

Total RNA of the tissues or cells was extracted in accordance with the Trizol Kit (Invitrogen Inc., Carlsbad, CA, USA), and mRNA was reversely transcripted into the first strand of cDNA using the Moloney murine leukaemia virus (MMLV) reverse transcriptase kit (Takara, Japan). qRT‐PCR was carried out using SYBR Green (Takara, Japan). The primers of *CXCR7*,* CXCR4, MMP‐2* and *glyceraldehyde‐3‐phosphate dehydrogenase* (*GAPDH)* are shown in Table 2. A standard two‐step PCR amplification process was performed as follows: 95°C pre‐degeneration for 30 sec.; 95°C for 5 sec. and 60°C for 34 sec. (40 cycles in total); and 72°C extension for 5 min. The change in the fluorescence signal was detected by a qPCR system. The fluorescence value at 60°C was measured as the relative quantity of the target genes. After the reaction, the results were automatically calculated and analysed by the computer. With GAPDH as an internal reference, the relative quantity was expressed as 2^−ΔΔCt^, in which Δ*C*_t_ = *C*_t_ value of the target gene – *C*_t_ value of *GAPDH*, and ΔΔ*C*_t_ = Δ*C*_t_ of each sample – Δ*C*_t_ of the sample in the blank control group. A mean value was obtained from each experiment after being performed in triplicate.

**Table 2:** Primer sequences

CXCR7	Forward: 5′‐TGCATCTCTTCGACTACTCAGA‐3′
	Reverse: 5′‐GGCATGTTGGCACATCAC‐3′
CXCR4	Forward: AAAATCTTCCTGCCCACCAT
	Reverse: ACGCCAACATAGACCACCTT
MMP‐2	Forward: 5′‐CGATGTCCAGCGAGTAGA‐3′
	Reverse: 5′‐TCACCTCATTGTATCTCCAGAA‐3′
GAPDH	Forward: 5′‐GGACCTGACCTGCCTCTAG‐3′
	Reverse: 5′‐GTAGCCCAGGATGCCCTTGA‐3′

CXCR7, CXC chemokine receptor 7; CXCR4, CXC chemokine receptor 4; MMP‐2, matrix metalloproteinase‐2; GAPDH, glyceraldehyde‐3‐phosphate dehydrogenase.

### Western blotting

After incubation, the cells were washed three times with phosphate‐buffered saline (PBS) and extracted with 100 μl/50 ml of lysis buffer per well for protein extraction. Also, the fresh tumour tissues temporarily stored in liquid nitrogen were homogenized in radio immunoprecipitation assay (RIPA) lysates. An equal amount of protein sample was added to the polyacrylamide gel, followed by electrophoretic separation under a voltage of 100 V. Next, the protein sample was transferred to a PVDF membrane, which was blocked with 10% milk for 2 hrs, and then immersed in diluted CXCR7 primary antibody (CXCR7 antibody: ab169946, 1:1000, Abcam Inc., Cambridge, MA, USA), CXCR4 primary antibody (CXCR4 antibody: ab1670, 1:1000, Abcam Inc.), MMP‐2 primary antibody (ab110186, 1:1000, Abcam Inc.), antivascular endothelial growth factor (VEGF) primary antibody (ab46154, 1:1000, Abcam Inc.) or GAPDH antibody (ab70699, 1:2000, Abcam Inc.) at 4°C overnight. The sample was then washed three times with Tris‐buffered saline–Tween 20 (TBST) and incubated with horseradish peroxidase(HRP)‐labelled secondary antibody (1:2000) (GenScript Co., Ltd., Nanjing, China) for 1 hr at room temperature, shaking at 20 r.p.m. Then, it was washed three times with TBST, immediately followed by visualization with an electrochemiluminescence (ECL) system for quantitative information. With GAPDH as an internal reference, the integral optical density ratio of the target protein to GAPDH was calculated. The experiments were repeated three times, and the mean values were obtained.

### MTT assay

The cells in the logarithmic growth phase were selected to prepare a cell suspension (10^5^/ml) in an RPMI 1640 culture medium containing 10% foetal bovine serum. The cell suspension was inoculated in a 96‐well culture plate (200 μl × 6 wells) for a 24‐ to 72‐hrs culture at 37°C with 5% CO_2_. 20 μl of MTT reagent (5 mg/ml, Sigma Company, St. Louis, MI, USA) was added per well, followed by an incubation for 4 hrs at 37°C with 5% CO_2_. After discarding the culture solution, each well had 150 μl of dimethyl sulfoxide (DMSO) added, followed by a 10‐min light shaking for crystal dissolution. The absorbance value of each well was detected using an enzyme‐linked immunoassay instrument at 0 hr, 24, 48 and 72 hrs later, respectively. An MTT curve was drawn with the absorbance values as the ordinate and the interval time as the abscissa. The experiment was repeated three times, and the mean values were obtained.

### Transwell assay

The Transwell chamber was applied for the measurement of the HCCLM3 cell invasion ability. Matrigel was diluted to 100 μg/ml using an RPMI 1640 culture medium containing 0.5% foetal bovine serum and then placed onto the membrane surface of a Transwell chamber (with a polyethylene terephthalate microporous membrane of 8‐μm pores between the upper and lower chambers). This was followed by a 30‐min. incubation at 37°C for gelling, and then serum‐free medium was added for balance overnight. The cells in the logarithmic growth phase were harvested and suspended in a serum‐free RPMI 1640 medium for density adjustment. A cell suspension (200 μl, about 5 × 10^5^) was placed into the upper chamber and RPMI 1640 culture medium (600 μl) containing stromal cell‐derived factor 1 (SDF‐1) (100 ng/ml) was added into the lower chamber for a 24‐hrs incubation at 37°C with 5% CO_2_. Following removal from the Transwell chamber, the cells on the membrane of the upper chamber were gently swabbed away, whereas the cells in the lower chamber were stained for 20 min. with 0.1% crystal violet. A microscope (× 100) was used to directly observe the cells passing through the membrane. Six fields were selected randomly to count and record the number of cells. The experiment was repeated three times, and the mean values were obtained.

### Establishment of a Buffalo rat model of HCC and grouping

A total of 60 SPF male 8‐week‐old Buffalo rats (mean weight of 220 ± 15 g) were obtained from the Animal Center, the Fifth Affiliated Hospital of Wenzhou Medical University, Lishui Hospital of Zhejiang University, Lishui Central Hospital. All rats were given standard rodent food and unlimited drinking water. The experimental programme was approved by the Ethics Committee of the Animal Experimental Center of the Fifth Affiliated Hospital of Wenzhou Medical University, Lishui Hospital of Zhejiang University, Lishui Central Hospital, and carried out in strict accordance with the recommendations in the *Guide for the Care and Use of Laboratory Animals of the National Institutes of Health* 22.

The well‐proportioned HCCLM3 cell suspension (0.5 ml, containing 2 × 10^6^ cells) was injected into the thigh muscle of the rats (*n* = 10) in order to obtain retransplantable tumours. At 14 days after injection, the subcutaneous tumours with a diameter over 1 cm were dipped in a serum‐free DMEM medium. The tumour capsule was removed with a scalpel, and the surrounding tumour tissue was immersed in sterile ice saline, rinsed twice and then cut into 1‐mm^3^ sized tumour pieces. The rats were administered with an intraperitoneal injection of anaesthesia (10% chloral hydrate, 1 ml/250 g) and the limbs were fixed in a supine position, followed by a midline abdominal shaving from the xiphoid, disinfection (with Anerdian) and a layer‐by‐layer opening along the linea alba until the abdominal cavity was exposed. The left lobe of the liver was immobilized to avoid its movement due to respiration. With the liver surface at an angle of 30 degrees, ophthalmic forceps were used to puncture the liver capsule and reach a depth of about 0.5 cm. Then, a cotton swab was used to apply pressure the puncture point until the bleeding stopped. After this, a tumour piece was implanted and, in the absence of continued bleeding, a layer‐by‐layer abdominal closure and disinfection were performed. After surgery, the rats were placed in a lateral position in the cages with soft light and a quiet environment. The room temperature was maintained at 25–30°C. Close attention was payed to the rats’ vital signs, such as respiration and heart rate.

All 50 HCC model rats were divided into five groups receiving different treatment regimens (10 rats in each group): the *CXCR7*‐shRNA + TACE group (receiving B‐mode ultrasound‐guided *CXCR7* RNA interference therapy combined with TACE), the *CXCR7*‐shRNA group (receiving B‐mode ultrasound‐guided *CXCR7* RNA interference therapy), the TACE group (treated with B‐mode ultrasound‐guided equivalent physiological saline combined with TACE), the negative control (NC) group (treated with B‐mode ultrasound‐guided equivalent empty vector, but not any therapy) and the control group (treated with B‐mode ultrasound‐guided equivalent physiological saline, but not therapy).

### Transfection under B‐mode ultrasonography (US) *in vivo*

On the 7th day after transplantation, presence of tumours in Buffalo rats was confirmed with magnetic resonance imaging (MRI). The tumour formation rate was 100%. The experiment was carried out on the 14th day after modelling. The tumour‐bearing rats were anaesthetized with an intraperitoneal injection of 10% chloral hydrate at a dose of 400 mg/kg, followed by skin preparation, sterilization and fixation in a supine position. The high‐frequency (frequency, 15 MHz; mechanical index, 0.88) linear‐array probe UST‐5410, which was used mainly for the superficial parts, was applied for horizontal and vertical exploration in the upper abdomen. An 80‐μl virus (5 × 10^8^ TU/ml) was diluted in 600 μl with a transfection enhancement solution containing polybrene (final concentration 5 μg/ml) and extracted into a 1‐ml syringe. This was followed by a B‐mode ultrasound‐guided percutaneous/transhepatic puncture into the tumour and injections at three loci at the tumour base and one locus above, with the four loci distributed as evenly as possible. The rats’ sleep, eating habits and weight were then closely monitored to determine whether they were appropriate for further experimental operation.

### Transcatheter arterial chemoembolization

After a 24‐hrs *in vivo* transfection, the HCC rats were treated with a retrograde intubation in the gastroduodenal artery. The rats were anaesthetized by an intraperitoneal injection of 10% perchlorate hydrate at a dose of 1 ml/250 g and then immobilized on the rat board, followed by abdominal depilation and disinfection and a layer‐by‐layer opening along the ventral midline. The gastroduodenal artery was exposed and isolated under a stereomicroscope, with the distal end ligated by a silk thread and the proximal end entangled by another silk thread. A short silk thread was placed between the two threads for catheter fixation. A catheter (outside diameter 0.64 mm and inside diameter 0.30 mm) with an oblique mouth was inserted to the gastroduodenal artery from a cut (about 2/3 the vascular diameter) and fixed using a retrograde method. A successful intubation was indicated by blood that flew into the catheter and formed a beating blood column. No resistance was found with an injection of normal saline. A successful intubation was followed by a transcatheter injection of lipiodol or normal saline at a speed of about 0.1 ml/3 min. Next, the proximal end of the gastroduodenal artery was ligated, followed by an extubation and layer‐by‐layer suture. In TACE, a selective separation and temporary occlusion of the hepatic artery under the microscope could prevent the embolic agent from entering back into the truncus coeliacus. Moreover, a blockade of the right hepatic artery can achieve hepatic segmental embolization to guarantee the survival rate of the rats after the experiment. After a successful intubation, the rats in the *CXCR7*‐shRNA + TACE and TACE groups were injected with a mixture of iodized oil and normal saline, whereas the rats in the *CXCR7*‐shRNA, NC and control groups were injected with normal saline only. Iodized oil and water were diluted at a ratio of 1:2; the total volume of the transcatheter injection of iodized oil or saline was 0.6 ml/kg (the weight of a rat).

### Observation of the survival time of the rats and specimen reservation

After TACE, measurements were performed with respect to their body weight every other day and the longest diameter of the tumour (L) weekly using B‐mode US. A ruler was placed perpendicular to L to obtain the shortest diameter (S). The tumour size was calculated according to the following formula: L × S^2^ × 1/2. At the same time, the rats were closely monitored for changes in eating, activity and hair colour. When severe dyscrasia was present as a result of a deteriorating state, the rats were killed *via* neck dislocation. Following the removal of necrotic tissues, the tumour was immediately placed into formaldehyde or liquid nitrogen for Western blot and immunohistochemistry analysis, respectively. Then, the chest was cut open along the midline for the observation of lung metastasis. The double lungs were removed, cleansed with normal saline, placed in formaldehyde and prepared for HE staining analysis.

### Immunohistochemistry staining

After removing the necrotic tissues, the fresh tumour specimens were cut to about 0.4 cm in thickness and placed in 10% formalin for a 24‐hrs fixation, followed by a conventional paraffin embedding. The wax block was cut into 3‐μm paraffin sections. The following procedures were performed according to the instructions of the immunohistochemical detection reagent kit (Aijie Biological Technology Co., Ltd., Changsha, China): dewaxing, alcoholic dehydration, blocking of endogenous peroxidase by 3% hydrogen peroxide, antigen retrieval, addition of primary antibodies (CXCR7 antibody: ab169946, 1:1000, Abcam Inc.; MMP‐2 antibody: ab86607, 1:1000, Abcam Inc.; VEGF antibody: ab46154, 1:100, Abcam Inc.; CD31 antibody: ab28364, 1:100, Abcam Inc.) for incubation, addition of biotin‐labelled secondary antibody for incubation, DAB coloration and haematoxylin counterstaining. Positive results were represented as the appearance of brown granules in the cytoplasm or nucleus. Two or three fields (×400) were analysed in the staining analysis. A total of 22 fields (×400) were analysed per group of rats. The expressions of CXCR7, MMP‐2 and VEGF were evaluated according to the percentage of immune‐reactive cells. Microvessel density (MVD) was detected and counted under a high‐power microscope. Any independent positive staining region of CD31 was counted as an independent vessel, and identified as the mean value of the microvessel count in each field (×400).

### H&E staining

Lung tissues of tumour‐bearing mice were collected for H&E staining. An optical microscope was used to calculate the number of pulmonary nodules. After tissue collection, the sections were embedded in paraffin, dewaxed in xylene (10 × 2 min.), immersed in tap water for 15 min. or in warm water (about 50°C) for 5 min. and rinsed with running water. Then, differentiation with hydrochloric acid ethanol was performed for 30 sec. (lifting and thrusting for several times), which was followed by immersion in tap water for 15 min. or in warm water (about 50°C) for 5 min., staining with haematoxylin–eosin solution for 2 min., dehydration in gradient ethanol, clearing in xylene (10 × 2 min) and sealing with neutral resin. Observation and photographing were made with the optical microscope.

### Statistical methods

SPSS 17.0 software was used for statistical analysis. Measurement data, which were expressed as mean ± standard deviation ($\overset{¯}{x}$ ± s), were analysed by one‐way analysis of variance for comparison of *MMP‐2* and tumour size among the groups, and LSD‐t was used for comparisons between two groups. Fisher's exact test was applied to determine the difference in lung metastasis in each group. A *P* value <0.05 was regarded as statistically significant.

## Results

### Identification of virus infection efficiency in HCCLM3 cells

**Figure 1:**
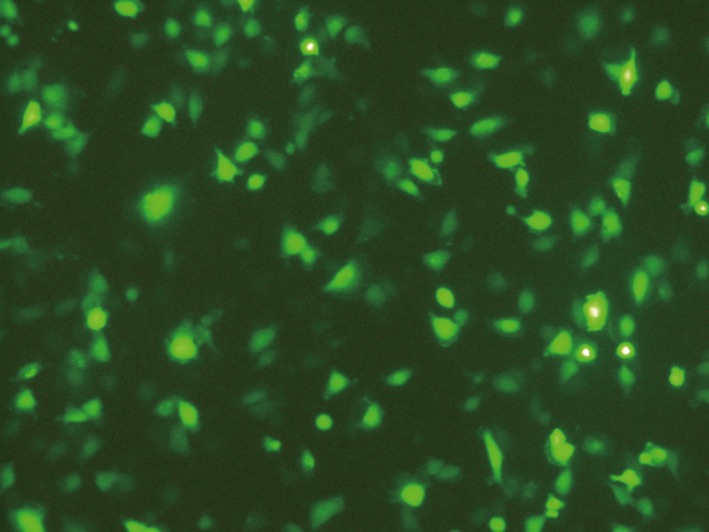
GFP expression in HCCCLM3 cells following transfection with LV‐CXCR7 shRNA (×400). Note: GFP, green fluorescent protein; shRNA, short hairpin RNA.

At 72 hrs after infection by LV‐*CXCR7*‐shRNA, the HCCLM3 cells were observed under an inverted fluorescence microscope for green fluorescent protein (GFP) expression, which was identified in the majority of the cells (Fig. 1), indicating that the cells grew well and LV‐*CXCR7*‐shRNA efficiently infected the HCCLM3 cells.

### Expressions of CXCR7, CXCR4 and MMP‐2 in HCCLM3 cells

**Figure 2:**
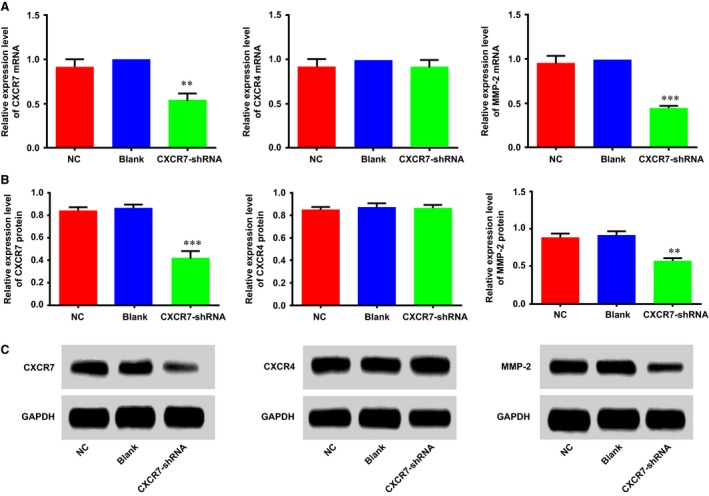
Changes of CXCR7, CXCR4 and MMP‐2 expression in HCCLM3 cells after transfection. (**A**) The histograms represent the relative mRNA expressions of CXCR7, CXCR4 and MMP‐2 in each group; (**B**,** C**) the histograms and Western blotting represent the comparisons of CXCR7, CXCR4 and MMP‐2 protein expressions among the groups. Note: ** represents *P* < 0.05 compared with the NC group and the blank group; *** represents *P* < 0.001 compared with the NC group and the blank group; CXCR7, CXC chemokine receptor 7; CXCR4, CXC chemokine receptor 4; MMP‐2, matrix metalloproteinase‐2.

Figure 2A shows the relative mRNA expressions (2^−ΔΔCt^ values) of *CXCR7*,* CXCR4* and *MMP‐2* in the three groups. Compared with the blank group, the *CXCR7*‐shRNA group showed significantly decreased mRNA expressions of *CXCR7* and *MMP‐2* in HCCLM3 cells (both *P* < 0.05), whereas the NC group showed no significant changes (both *P* > 0.05). No differences were detected in the mRNA expression of *CXCR4* among the groups (*P* > 0.05). We simultaneously measured the protein expressions of CXCR7, CXCR4 and MMP‐2 in HCCLM3 cells of the three groups by Western blotting and identified similar findings that the *CXCR7*‐shRNA group showed significantly lower protein expressions of CXCR7 and MMP‐2 than the NC group (*P* < 0.001); however, no differences were found in the protein expressions of CXCR7 and MMP‐2 between the blank group and the NC group (*P* > 0.05). The protein expression of CXCR4 in each group showed no evident differences (Fig. 2B,C).

### Effects of CXCR7‐shRNA on the proliferation and invasion of HCCLM3 cells

**Figure 3:**
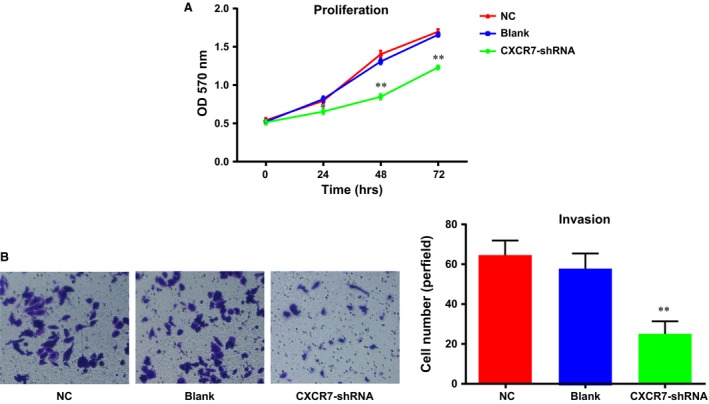
Influences of CXCR7‐shRNA on the proliferation and invasion of HCCLM3 cells after transfection. (**A**) Growth curves of HCCLM3 cells in each group; (**B**) the invasion ability of HCCLM3 cells in each group. Note: ** represents the *P* < 0.05 when compared with the NC group and the blank group; CXCR7, CXC chemokine receptor 7.

MTT experiments were performed in the HCCLM3 cells of each group, and the optical density (OD) value was detected at 0, 24, 48 and 72 hrs, respectively. These data were utilized to draw a growth curve with the time‐points for the horizontal coordinates and the OD values for the vertical coordinates (Fig. 3A). As shown in the figure, starting from the time‐point of 24 hrs, the cell proliferation ability of the cells in the *CXCR7*‐shRNA group was significantly decreased compared with the Con and NC groups (*P* < 0.01). However, no significant difference was found in the cell growth curve of the NC and blank groups (*P* > 0.05). The invasion ability of the cells in each group was detected by Transwell invasion experiments, and the results (Fig. 3B) showed that the number (24.76 ± 6.26) of HCCLM3 cells passing through the chambers in the *CXCR7*‐shRNA group was significantly lower than that in the NC and blank groups, 58.64 ± 7.58 and 64.58 ± 7.34, respectively. This demonstrated a significantly decreased invasion ability of HCCLM3 cells in the *CXCR7*‐shRNA group (*P* < 0.01). The NC and blank groups showed no significant difference in the invasion ability of HCCLM3 cells (*P* > 0.05).

### Effect of CXCR7‐shRNA on body weight of HCC rats after TACE

**Figure 4:**
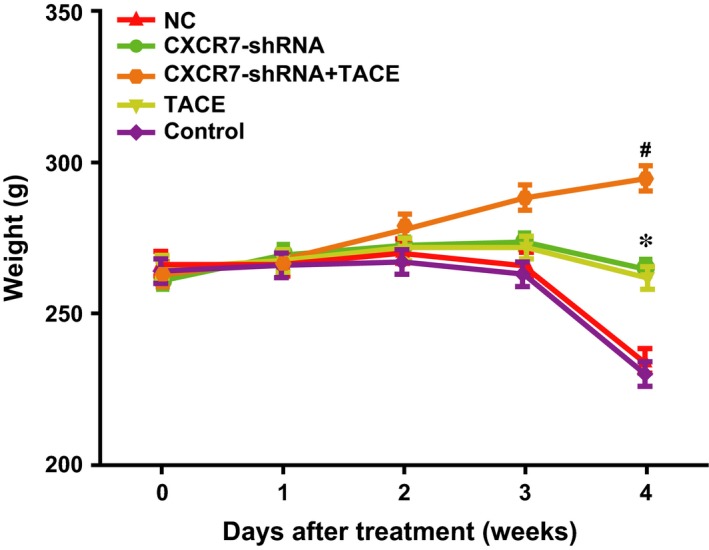
Effect of CXCR7‐shRNA on body weight of HCC rats after TACE. Note: * represents comparison with the control group, *P* < 0.05; # represents comparison with the TACE group, *P* < 0.05; TACE, transcatheter arterial chemoembolization; CXCR7, CXC chemokine receptor 7; shRNA, short hairpin RNA.

The rats did not display obvious weight gain or loss in the first week after TACE. In the second week, the rats in all groups began to gain weight. In the third week, the rats in the *CXCR7*‐shRNA + TACE group showed a continuous weight gain, whereas the rats in the control and NC groups showed a significant decrease in weight. This can be a result of tumour growth. The rats in the *CXCR7*‐shRNA and TACE groups showed limited weight gain or loss. In the fourth week, with the exception of the continuous weight gain of the rats in the *CXCR7*‐shRNA + TACE group, weight loss was identified in the other three groups of rats (all *P* < 0.05), and a significant weight loss was identified in the control group (compared with the *CXCR7*‐shRNA group, *P* < 0.01; compared with the TACE group, *P* < 0.05) (Fig. 4).

### Effects of CXCR7‐shRNA on the survival time and tumour growth in HCC rats after TACE

**Figure 5:**
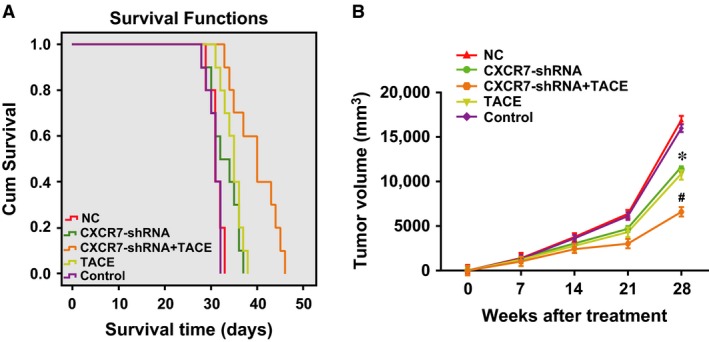
Effects of CXCR7‐shRNA on the survival time and tumour growth of HCC rats after TACE. (**A**) the survival time of HCC rats in each group; (**B**) the changes of orthotopic tumour size in HCC rats in each group (orthotopic tumour size was measured weekly). Note: * represents the *P* < 0.05 compared with the control group; ^#^ represents the *P* < 0.05 compared with the TACE group; CXCR7, CXC chemokine receptor 7; TACE, transcatheter arterial chemoembolization.

The median survival time of the rats in each group after treatment was as follows: 40 days (95% CI: 35.45–44.55 days) for the *CXCR7*‐shRNA + TACE group, 33 days (95% CI: 27.35–36.65 days) for the *CXCR7*‐shRNA group, 35 days (95% CI: 33.48–36.52 days) for the TACE group (receiving TACE treatment only), 31 days (95% CI: 29.48–32.52 days) for the NC group and 31 days (95% CI: 29.99–32.01 days) for the control group. As indicated by the Kaplan–Meier results (Fig. 5A), there were significant differences in the survival time of the rats in each group (χ^2^ = 36.985, *P* < 0.0001), with the longest survival time in the *CXCR7*‐shRNA + TACE group, followed by the *CXCR7*‐shRNA, TACE and NC groups. The shortest survival time was in the control group, indicating that RNA interference of *CXCR7* improved the TACE efficiency in the treatment of HCC by prolonging the survival time of the rats.

Figure 5B shows the follow‐up results of the tumour growth of the rats in each group after treatment using B‐mode US as follows: tumour growth was suppressed in the *CXCR7*‐shRNA + TACE group; tumour growth was slightly inhibited in the *CXCR7*‐shRNA and TACE groups; and tumours grew rapidly in the control group. Four weeks after the treatment, the tumour size in the *CXCR7*‐shRNA + TACE group was significantly smaller than in the other three groups (all *P* < 0.01), and the sizes in the *CXCR7*‐shRNA and TACE groups were significantly smaller than in the control group (both *P* < 0.01). Therefore, RNA interference mediated by *CXCR7*‐shRNA improved the TACE efficiency for the treatment of HCC by inhibiting tumour growth.

### Effects of CXCR7‐shRNA on lung metastasis of HCC rats after TACE

**Figure 6:**
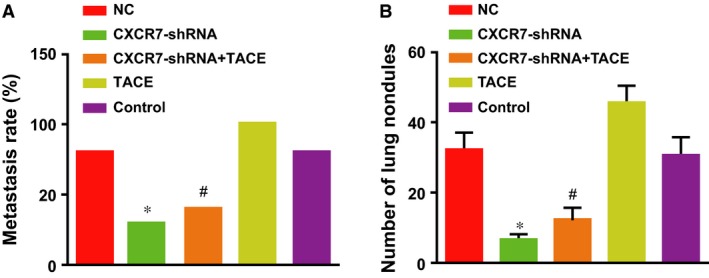
Effects of CXCR7‐shRNA on lung metastasis of HCC rats after TACE. (**A**) Lung metastasis rate; (**B**) number of lung metastatic nodules. Note: * represents comparison with the control group, *P* < 0.05; # represents comparison with the TACE group, *P* < 0.05; CXCR7, CXC chemokine receptor 7; TACE, transcatheter arterial chemoembolization; HCC, hepatocellular carcinoma.

As shown in Figure 6A, the rate of lung metastasis decreased from 80% (8/10) in the control group and the NC group to 30% (3/10) in the *CXCR7*‐shRNA group, and from 100% (10/10) in the TACE group to 40% (4/10) in the *CXCR7*‐shRNA + TACE group, demonstrating that depletion of CXCR7 reduced lung metastases caused by TACE. A significant difference was shown in the rate of lung metastasis between the two groups with TACE (the *CXCR7*‐shRNA + TACE and TACE groups) (*P* = 0.003). The average number of lung nodules of the rats was highest in the TACE group compared with the other three groups (all *P* < 0.01). The number decreased significantly in the *CXCR7*‐shRNA + TACE and *CXCR7*‐shRNA groups compared with the TACE and control groups (all *P* < 0.01) (Fig. 6B). Thus, RNA interference of *CXCR7* effectively inhibited lung metastasis caused by the tumour or TACE.

### Effects of CXCR7‐shRNA on the expressions of CXCR7, MMP‐2 and VEGF and CD31‐positive vessel count of HCC rats after TACE

**Figure 7:**
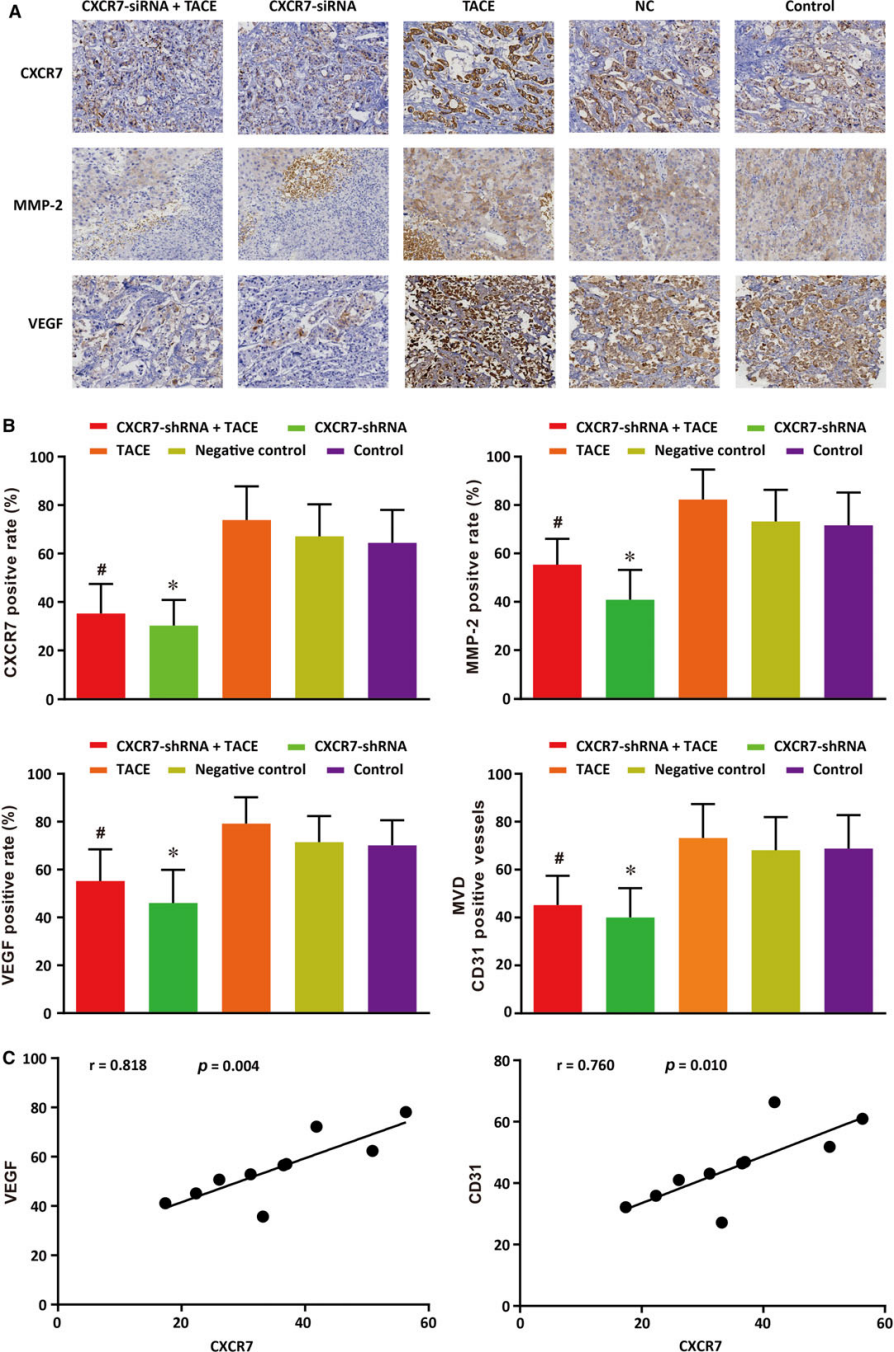
Effects of CXCR7‐shRNA on the positive expressions of CXCR7, MMP‐2 and VEGF and CD31 in tumour tissues of rats with HCC after TACE.Note: A. The positive staining of CXCR7, MMP‐2 and VEGF; B. The positive rates of CXCR7, MMP‐2 and VEGF and CD31; C. Correlations between CXCR7 and VEGF and between CXCR7 and CD31. CXCR7, CXC chemokine receptor 7; MMP‐2, matrix metalloproteinase‐2; VEGF, vascular endothelial growth factor; TACE, transcatheter arterial chemoembolization; HCC, hepatocellular carcinoma.

The expressions of CXCR7, MMP‐2, VEGF and CD31, the indexes of tumour metastasis, were determined by immunohistochemical analysis of tumour tissues. As seen in Figure 7A, CXCR7 was mainly expressed on the cell membrane, with some expression in the cytoplasm. MMP‐2 and VEGF were also mainly expressed in cytoplasm. As demonstrated in Figure 7B, the positive staining percentages of CXCR7, MMP‐2 and VEGF, as well as the CD31‐positive vessel count in each field, were the highest in the TACE group. The percentages and the vessel count decreased significantly in the *CXCR7*‐shRNA + TACE and *CXCR7*‐shRNA groups when compared with the TACE and control groups (all *P* < 0.05). No differences were found in the positive staining percentage or the vessel count between the NC group and the control group. Therefore, we concluded that the knock‐down of *CXCR7* significantly inhibited the elevation of CXCR7, MMP‐2, VEGF and CD31 induced by TACE. The analysis for the correlations of CXCR7 with VEGF and CD31 revealed that CXCR7 was positively correlated with both VEGF and CD31 expressions (*r* = 0.818, *P* = 0.004; *r* = 0.760, *P* = 0.010) (Figure 7C).

## Discussion

A vector expressing shRNA can be used to stably suppress gene expression in cell lines 23. In this study, we constructed a *CXCR7*‐shRNA lentiviral vector to stably silence *CXCR7* in HCCLM3 cells and investigate changes in the growth and metastasis. This lentiviral vector system facilitated improved transfection efficiency 24. Four different shRNA plasmids were constructed and fully verified by sequencing, which guaranteed that at least one plasmid could provide more than 40% gene expression inhibition (www.origene.com).

An MTT analysis was performed to determine the proliferation ability changes of HCCLM3 cells after the silencing of *CXCR7*. The results demonstrated that the proliferation ability of HCCLM3 cells was significantly weakened when undergoing *CXCR7* expression inhibition in HCCLM3 cells, which indicates that *CXCR7* played an important role in the promotion of HCCLM3 cell growth. This finding was consistent with previous reports of *CXCR7* promoting breast cancer and prostate cancer cell growth 12, 25. However, the mechanism remains elusive and will be further investigated in future research.

The results indicated that the number of migrated cells in the *CXCR7*‐shRNA interference group was significantly decreased when compared with the control group, which suggests that *CXCR7* acted as an important regulator in the invasion of HCCLM3 cells. The mechanisms of *CXCR7* on tumour invasion regulation remain controversial. However, previous studies have suggested that CXCR7 mediates tumour cell invasion through a mechanism involving the secretion of extracellular matrix proteases (MMPs) which are conducive to tumour cell invasion 25, 26. An essential initial step in cancer cell invasion is the interaction of tumour cells with extracellular matrix (ECM) components and basement membranes, specifically the degradation of the ECM by MMPs, such as MMP‐3, proteolytically cleaved, and activated by *MMP‐2*,* MMP*‐3, *MMP*‐13 and plasmin, which facilitates tumour cell dissemination 14, 25, 27. MMPs, which comprise a family of 23 endopeptidases, contribute to the degradation of all protein components of tissue extracellular matrices and basement membranes 28. Consequently, we suggest that the knock‐down of *CXCR7* expression inhibited the invasion ability of HCCLM3 cells *via* the suppression of *MMP‐2* expression.

We determined that in the 4th week after TACE, the rats in the *CXCR7*‐shRNA + TACE group continued to gain weight, whereas the rats in the *CXCR7*‐shRNA, TACE and control groups decreased in weight. Interestingly, the silencing of *CXCR7* significantly suppressed tumour growth. Previous studies have demonstrated that *CXCR7* plays an essential role in tumour growth 12, 26. Consistent with our findings, Zheng *et al*. 14 demonstrated that the knock‐down of *CXCR7* expression resulted in the inhibition of tumour growth in xenograft models of HCC. They suggested that the knock‐down of *CXCR7* inhibited the secretion of VEGF, one of the most prominent angiogenic factors produced by various tumour cells, and tube formation and thus regulated angiogenesis and tumour growth in HCC.

The rates of lung metastasis were 100%, 40% and 30% in the TACE, *CXCR7*‐shRNA + TACE and *CXCR7*‐shRNA groups, respectively, which demonstrated that RNA interference of *CXCR7* reduced lung metastases caused by TACE. The finding was partially in accordance with a recent report 14. Moreover, according to Xue *et al*. 8, the silencing of *CXCR7* inhibited the growth and lung metastasis of HCCLM3 tumours in nude mice. One study demonstrated that in an osteoarthritis model, the overexpression of *CXCR7* in chondrocyte cells induced increased expressions of interleukin‐8, osteopontin, matrix metalloproteinases‐2 and vascular endothelial growth factor, which suggests that *CXCR7* potentially affected the metastasis of HCC based on the close correlations between these genes and the metastasis of HCC 29. The tumour size was significantly smaller in the *CXCR7‐*shRNA group compared with the other groups. Thus, the reduced lung metastasis following depletion of CXCR7 could be attributed to tumour growth inhibition.

In the present study, we also identified the longest survival time of the rats in the *CXCR7*‐shRNA + TACE group, followed by the *CXCR7*‐shRNA (only) and TACE (only) groups, which suggested the effects of CXCR7 depletion and TACE therapy. According to Neve *et al*., *CXCR7* was considered a valuable prognostic indicator for lung metastasis and poor prognosis in HCC. However, no prognostic correlation of CXCR7 expression with overall survival was shown, with one potential explanation being that high *CXCR7* expression was regulated by CXCR4‐CXCL12 levels 30. We also suggest that the result was related to the tumour growth inhibition *via CXCR7* down‐regulation.

In conclusion, the present study demonstrated that CXCR7‐shRNA can inhibit the proliferation and invasion of HCC cells by down‐regulating CXCR7 and MMP‐2 expressions. CXCR7‐shRNA can also suppress tumour invasion and metastasis to improve the efficacy of TACE for HCC treatment by reducing the expressions of CXCR7, MMP‐2 and VEGF. The current findings suggest the potential therapeutic benefits of *CXCR7* as a novel molecular target for HCC. Therefore, further studies with more HCC cells are needed to verify our results.

## Conflict of interest

The authors have declared that no conflict of interest exist.
